# Prognostic Factors in Uterine Sarcoma Based on the Tumor Size Stratification: A Retrospective Study

**DOI:** 10.7759/cureus.65819

**Published:** 2024-07-31

**Authors:** Fumio Asano, Tohru Morisada, Mai Momomura, Hiromi Shibuya, Hironori Matsumoto, Yoichi Kobayashi

**Affiliations:** 1 Department of Obstetrics and Gynecology, Kyorin University School of Medicine, Tokyo, JPN

**Keywords:** sarcoma, prognosis, tumor, uterus, chemotherapy

## Abstract

Objective: Uterine sarcoma is a rare malignant gynecological tumor with a poor prognosis. Many studies have identified the clinical stage as an important prognostic factor; however, the heterogeneity of patient distribution in the International Federation of Gynecology and Obstetrics (FIGO) stage has reportedly required further revision. Therefore, this study retrospectively investigated the factors related to the prognosis of uterine sarcoma, with particular attention to tumor size, which can be evaluated preoperatively.

Methods: Clinical data were extracted from the medical records of patients with uterine sarcoma treated between January 2010 and January 2023. Kaplan-Meier survival curves were plotted according to clinical factors such as histological type, clinical stage, chemotherapy, and tumor size. Factors that were significant in the univariate analysis were subjected to the multivariate analysis using Cox proportional hazards regression.

Results: Thirty-four patients with uterine sarcoma, comprising 24 (70.5%), five (14.7%), three (8.8%), and two (5.9%) with leiomyosarcoma, undifferentiated sarcoma, high-grade endometrial stromal sarcoma, and low-grade endometrial stromal sarcoma, respectively, were included. Based on the FIGO stage, 15 (44.1%), six (17.6%), three (8.8%), and 10 patients had stage I, II, III, and IV disease, respectively, at the time of diagnosis. All patients underwent surgery as initial treatment; 15 received postoperative chemotherapy. Among the 32 patients with uterine leiomyosarcoma, undifferentiated sarcoma, or high-grade endometrial stromal sarcoma, overall survival differed significantly in the univariate analysis based on disease stage (I + II vs. III + IV) and tumor size (≤10 vs. >10 cm). However, only tumor size was an independent prognostic factor in the multivariate analysis.

Conclusion: Tumor size (≤10 vs. >10 cm) may possibly have a prognostic impact on uterine sarcoma.

## Introduction

Uterine sarcoma is a malignant mesenchymal tumor of the uterine corpus, accounting for 3-7% of all uterine malignancies. Uterine sarcoma has a poor prognosis, with a five-year survival rate of 17-55% [1,2]. Leiomyosarcoma is the most common histological type of uterine sarcoma, followed by endometrial stromal and undifferentiated uterine sarcoma [3].

Leiomyosarcomas arise from the smooth muscle of the uterus and can metastasize hematogenously, even in cases of early-stage lesions, with a recurrence rate of 45-80%; the most common metastatic sites are the lungs and liver [4,5]. Endometrial stromal tumors are classified as low- or high-grade tumors. Low-grade endometrial stromal tumors are generally more common in patients in their 40s; although these tumors are less malignant than other types, late recurrence may occur [6]. High-grade endometrial stromal sarcomas are stromal tumors with intermediate malignancy between conventional low-grade endometrial stromal sarcomas and undifferentiated sarcomas. Furthermore, they have a lower five-year survival rate than low-grade endometrial stromal sarcomas [6]. Undifferentiated sarcomas have a markedly poor prognosis; approximately 60% of patients with undifferentiated sarcomas have stage III or IV disease [6].

In general, patients with uterine sarcomas have poor prognoses; however, several reports have shown that complete surgical resection, radiation therapy, and chemotherapy may improve prognoses [3,7]. Furthermore, many studies have reported prognostic factors for uterine sarcoma, with clinical stage being an important factor [8-12]. Prior to the current International Federation of Gynecology and Obstetrics (FIGO) 2008 classification, the FIGO 1988 classification did not consider tumor size as a staging variable [13]. Subsequent reports demonstrated the usefulness of stratification by tumor size; thus, the FIGO 2008 classification considered a tumor size of 5 cm as a criterion size for stage I disease [2]. However, only 10-23% of uterine sarcomas have a tumor diameter ≤5 cm, with the median diameter being 7-12 cm, suggesting the need for a new size cutoff value of >5 cm [14-16]. Furthermore, Yim et al. [17] reported that the 2008 FIGO classification more accurately reflects the prognosis of early-stage (I + II) and advanced-stage (III + IV) than the 1988 FIGO classification, while Tan et al. [18] indicated that although FIGO 2008 has a useful subclassification for stage I with the introduction of tumor size, the heterogeneity of patient distribution in stages II-IV suggested further revision.

Therefore, this study retrospectively reviewed the clinical information of patients diagnosed with mesenchymal malignancies of the uterus after the initial surgical treatment to explore prognostic factors. Particularly, we reexamined the criterion of tumor size, which can be estimated preoperatively and may be related to prognosis.

## Materials and methods

Data on patient age, histological type, clinical stage, tumor size, adjuvant chemotherapy, chemotherapy regimens, and outcomes such as recurrence and death were retrospectively reviewed from the medical records of patients diagnosed with uterine mesenchymal malignancies after initial treatment between January 2010 and January 2023 at our institution. Chemotherapy regimens used were doxorubicin (Adriamycin, or ADM), docetaxel-ifosfamide-cisplatin (DIP), gemcitabine-docetaxel (GD) or ifosfamide-doxorubicin (IA). Patients with carcinosarcoma or adenosarcoma were excluded based on their treatment methods and FIGO 2008 stage.

This study was approved by the Ethics Committee, Kyorin University (approval number: R04-088), and the requirement for informed consent was waived owing to the study’s retrospective nature. This study followed the STrengthening the Reporting of OBservational studies in Epidemiology (STROBE) reporting guidelines.

Statistical analyses were performed using Statistical Package for the Social Sciences (IBM SPSS Statistics for Windows, IBM Corp., Version 29.0, Armonk, NY). Categorical data were presented as counts and percentages and analyzed using the chi-squared or Fisher’s exact tests, as appropriate. Survival analyses were performed using the Kaplan-Meier method, and the log-rank test was used to assess survival rate differences. Overall survival (OS) and progression-free survival (PFS) were also assessed for histological type, clinical stage, chemotherapy, and tumor size. We grouped FIGO stages I and II into the early-disease group and stages III and IV into the advanced-disease group for the survival analyses because of the small sample size. In this study, the tumor size was measured using a preoperative pelvic MRI, with the maximum diameter measured sagittal or axial or coronal on T2-weighted images. All P-values were two-sided, and a P-value <0.05 was considered significant.

## Results

We identified 34 patients with uterine sarcoma. The median age was 59.7 (range, 39-79) years. Histologically, 24 (70.5%), five (14.7%), three (8.8%), and two (5.9%) patients had leiomyosarcoma, undifferentiated sarcoma, high-grade endometrial stromal sarcoma, and low-grade endometrial stromal sarcoma, respectively. At the time of diagnosis, 15 (44.1%), six (17.6%), three (8.8%), and 10 patients (29.4%) had stage I, II, III, and IV disease, respectively, based on the FIGO staging guidelines (Table 1).

**Table 1:** Characteristics of the study population ADM: doxorubicin (Adriamycin); DIP: docetaxel-ifosfamide-cisplatin; FIGO: International Federation of Gynecology and Obstetrics; GD: gemcitabine-docetaxel; IA: ifosfamide-doxorubicin

Characteristics	Description	N = 34 (%)
Age (in years): mean (range)		59.7 (39-79)
Histological type	Leiomyosarcoma	24 (70.5)
	Undifferentiated sarcoma	5 (14.7)
	High-grade endometrial stromal sarcoma	3 (8.8)
	Low-grade endometrial stromal sarcoma	2 (5.9)
FIGO stage	Ⅰ	15 (44.1)
	Ⅱ	6 (17.6)
	Ⅲ	3 (8.8)
	Ⅳ	10 (29.4)
Tumor size	≤5 cm	3 (8.8)
	5 cm-10 cm	7 (20.6)
	>10 cm	24 (70.6)
Adjuvant chemotherapy	Yes	16 (47.1)
	No	18 (52.9)
Chemotherapy regimens	DIP	7 (43.7)
	GD	4 (25.0)
	ADM	3 (18.8)
	IA	1 (6.3)
	Pazopanib	1 (6.3)
Recurrence or progression	Yes	22 (64.7)
	No	12 (35.3)

All patients underwent surgery as the initial treatment. Sixteen patients received adjuvant chemotherapy; seven patients received DIP, four received GD, three received ADM, one received IA, and one received pazopanib.

Overall, 22 patients (64.7%) experienced disease recurrence or progression. The main metastatic sites were the lungs (n = 10), pelvis (n = 8), and liver (n = 4). Bones and lymph nodes were less common metastatic sites (n = 2 each). The median OS (n = 34) was 21 months (95% confidence interval (CI): 3.7-34.9), and the median PFS was 12 months (95% CI: 2.8-21.2).

Next, we focused on the prognosis in 32 cases of uterine leiomyosarcoma, undifferentiated sarcoma, and high-grade endometrial stromal sarcoma, which are histological types with a high malignant potential.

**Figure 1:**
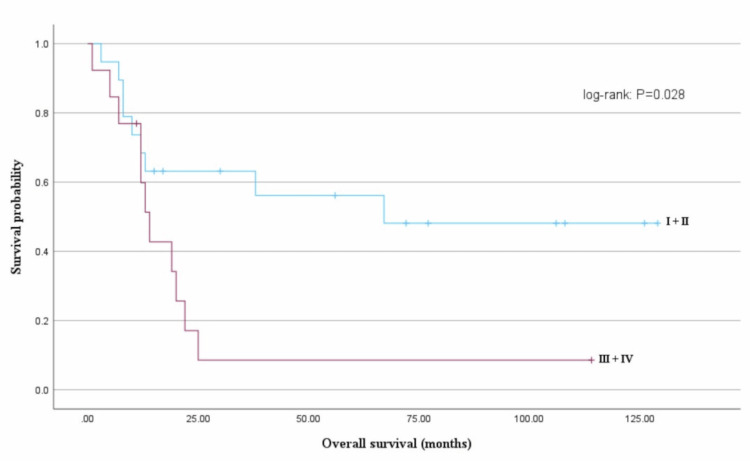
Overall survival (OS) and progression-free survival (PFS) based on the International Federation of Gynecology and Obstetrics (FIGO) staging OS based on FIGO stage groups (early-stage (I + II) vs. advanced-stage (III + IV)); the median OS significantly differed between early-stage (I + II) and advanced-stage (III + IV) disease (67 months vs. 14 months, log-rank test: P = 0.028).

Age (<51 years vs. ≥51 years) did not affect OS (68 months vs. 48 months, log-rank test: P = 0.355). Furthermore, the FIGO stage (stages I, II, III, and IV) and the median OS (stage I, not reached; stage II, 12 months; stage III, 20 months; and stage IV, 12 months) were not correlated (log-rank test: P = 0.128). However, the median OS differed significantly between early-stage (I + II) and advanced-stage (III + IV) disease groups (67 months vs. 14 months, log-rank test: P = 0.028) (Figure 1).

**Figure 2:**
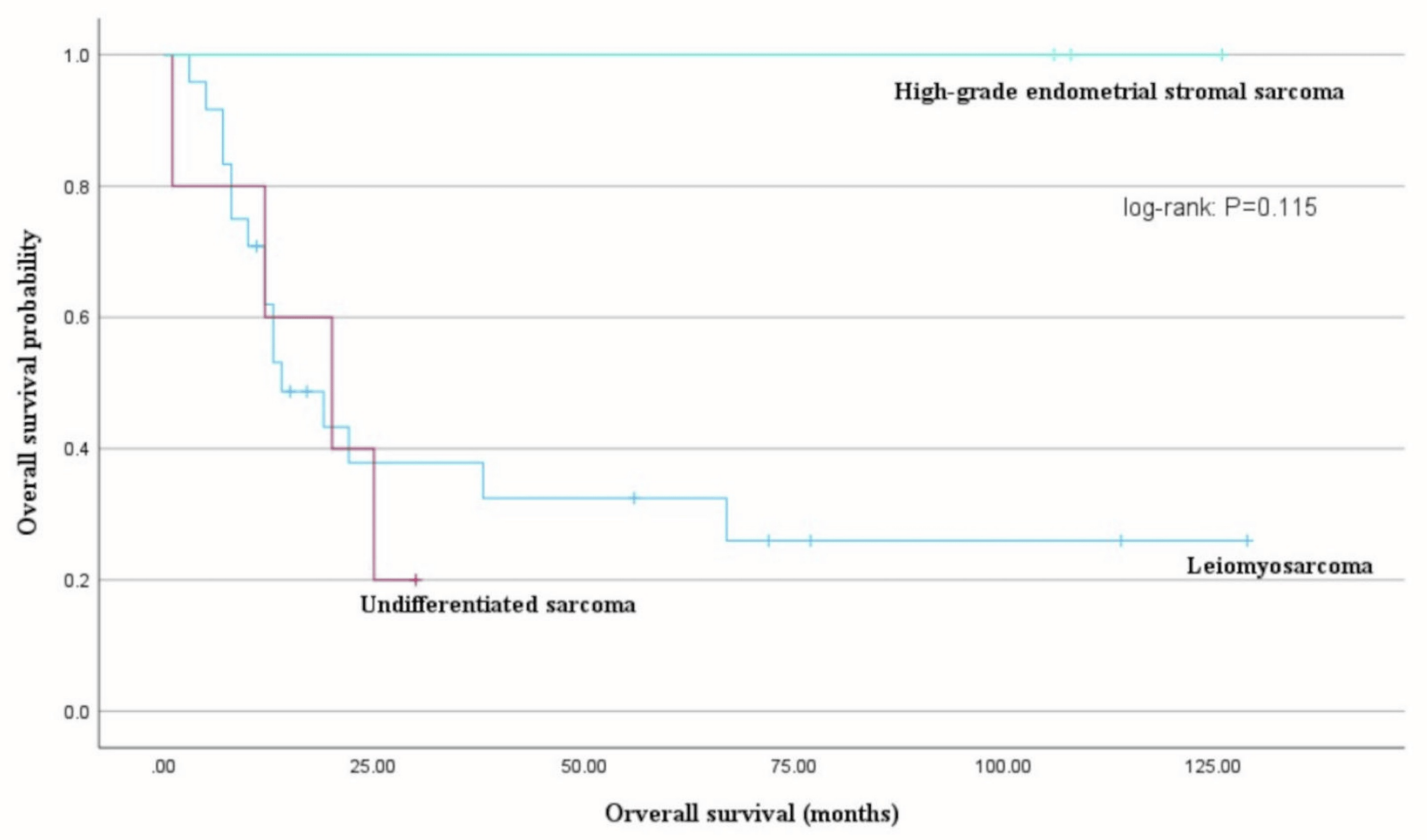
Overall survival (OS) based on the histological type OS did not differ based on the histological type (uterine leiomyosarcoma, undifferentiated sarcoma, or high-grade endometrial stromal sarcoma; log-rank test: P = 0.115).

The median PFS was longer for those in the early-stage disease group than for those in the advanced-stage disease group; nevertheless, the difference was not significant (30 months vs. 7 months, log-rank test: P = 0.067). OS did not differ based on the histological type (uterine leiomyosarcoma, undifferentiated sarcoma, or high-grade endometrial stromal sarcoma; log-rank test: P = 0.115) (Figure 2).

**Figure 3:**
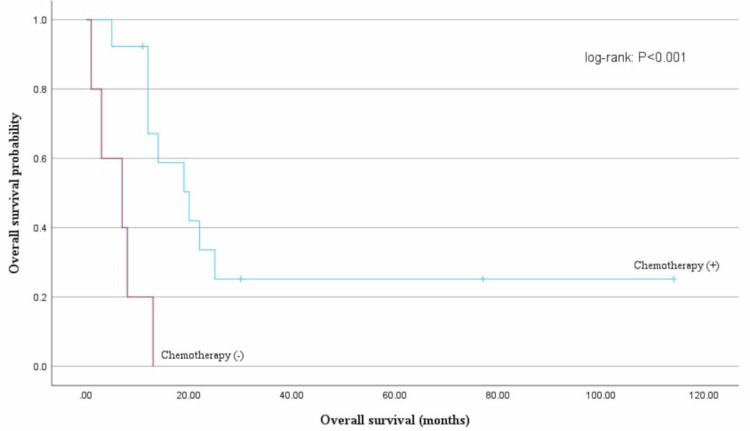
Overall survival (OS) based on chemotherapy OS based on stages II-IV groups; the median OS significantly differed between the chemotherapy group and the chemotherapy-native group (20 months vs. 7 months, log-rank test: P < 0.001).

The efficacy of adjuvant chemotherapy for stage I remains controversial. In this study, the efficacy of postoperative chemotherapy for stages II-IV was investigated. Patients with stages II-IV disease had a median OS of 20 months in the chemotherapy group compared with an OS of seven months in the chemotherapy-naive group, which indicated a significantly longer OS for the chemotherapy group (log-rank test: P< 0.001) (Figure 3).

**Figure 4:**
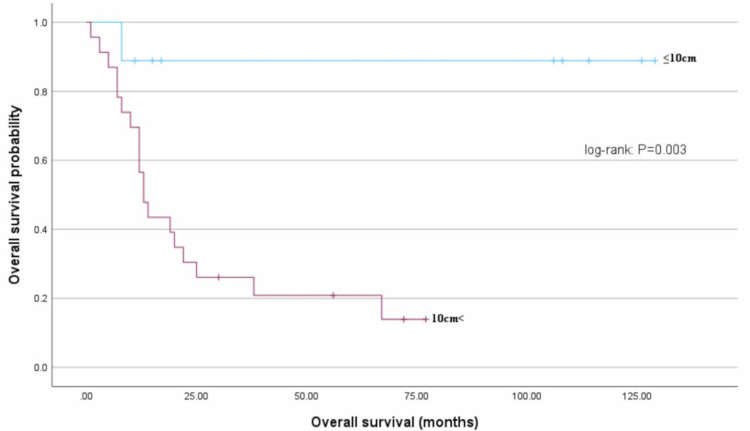
Overall survival (OS) based on tumor size Patient OS based on the tumor size (≤10 and >10 cm); the mean OS significantly differed between the ≤10 and >10 cm groups (115 months vs. 26 months, log-rank test: P = 0.003).

Next, the relationship between tumor size and prognosis in uterine sarcomas was investigated. The mean OS of the group with tumors ≤10 cm was 115 months, which was significantly longer than the mean OS of 26 months for the group with tumors >10 cm (log-rank test: P = 0.003) (Figure 4).

**Figure 5:**
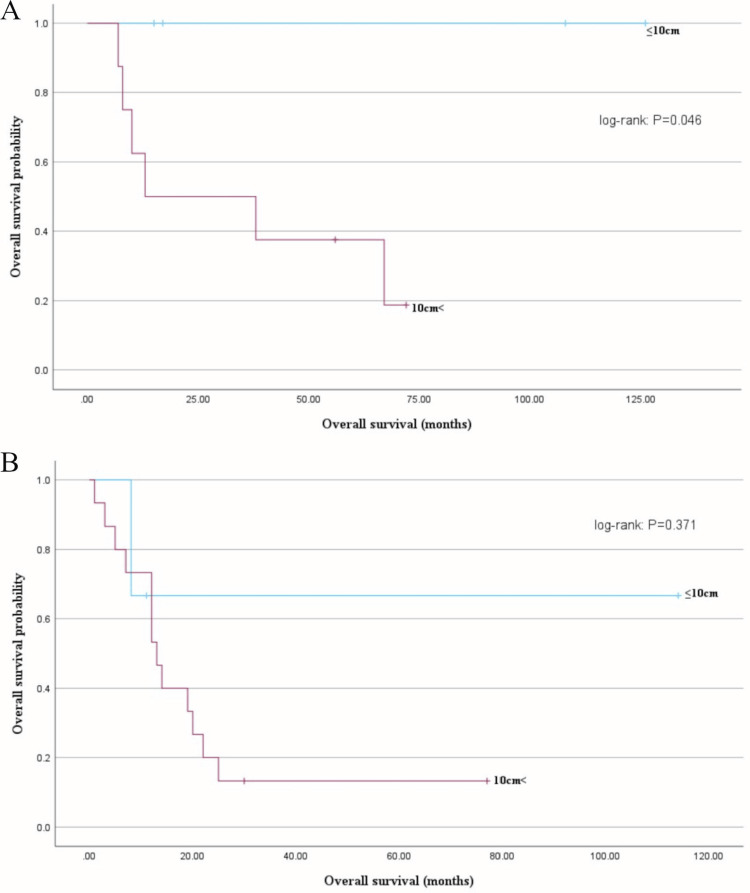
Overall survival (OS) based on tumor size by FIGO stage (I and II-IV) (A) Patient OS based on stage I groups; OS significantly differed between the tumor size (≤10 and >10 cm) (log-rank test: P = 0.046). (B) Patient OS based on stages II-IV groups; the mean OS did not significantly differ between the tumor size (≤10 and >10 cm) (78 months vs. 21 months, log-rank test: P = 0.371).

When examined by clinical stage, there were no deaths in the group of patients with stage IB with tumors ≤10 cm, and the mean OS for this group was significantly longer than that of the group with tumors >10 cm (66 months vs. 33 months, log-rank test: P = 0.0.046) (Figure 5A). In contrast, comparing the group of patients with stages II-IV tumors, the mean OS of the group with tumors ≤10 cm was 78 months, whereas that of the group with tumors >10 cm was 21 months; although not significant, this comparison indicated a trend toward longer OS in the group with tumors ≤10 cm (log-rank test: P = 0.371) (Figure 5B).

The multivariate analysis included age, tumor stage (I + II and III + IV), and tumor size (≤10 cm and >10 cm). Only the ≤10-cm and >10-cm tumor size groups had a significant effect (hazard ratio: 10.948; 95% CI: 1.378-86.442) (Table 2).

**Table 2:** Multivariate Cox hazard model analysis for factors associated with the overall survival of patients with uterine sarcoma CI: confidence interval; HR: hazard ratio

Factors	B	Wald	HR (95% CI)	P-value
Age (years)	-0.009	0.258	0.991 (0.959-1.025)	0.611
Tumor stage (I + II and III + IV)	0.751	2.284	2.118 (0.800-5.606)	0.131
Tumor size (≤10 cm and >10 cm)	2.393	5.153	10.948 (1.378-86.443)	0.023

## Discussion

Uterine sarcoma is a rare malignancy with a poor prognosis. Patient age, tumor size, histological type, mitotic index, nuclear atypia, and clinical stage are reported prognostic factors for uterine sarcoma. However, the results vary between studies, partly because of the rarity and heterogeneity of uterine sarcomas [8-12,14-16,19-21]. According to a 2012 systematic review that analyzed data from 1970 to 2011, uterine leiomyosarcoma was the most common uterine sarcoma (63%), followed by endometrial stromal sarcoma (21%), and undifferentiated uterine sarcoma (5%) [3]. The results of our study are consistent with these findings. Furthermore, many studies have indicated that the clinical stage is an important prognostic factor for uterine sarcoma [8-12]. For example, Kapp et al. [8] reported the five-year survival rates for uterine leiomyosarcoma as follows: stage I, 75.8%; stage II, 60.1%; stage III, 44.9%; and stage IV, 28.7%. This study also found significant differences between early-stage and advanced cases, suggesting that stage is an important prognostic factor.

Surgical treatment is the only treatment for uterine sarcoma that improves prognosis, and the effects of chemotherapy or radiation therapy on prognosis remain controversial [3,7,16,22-26]. Several previous studies report that chemotherapy may or may not be effective for stage I tumors [16,27]. The 2023 National Comprehensive Cancer Network Guidelines state that adjuvant chemotherapy should be considered as postoperative therapy for stage II and higher uterine sarcomas [21]. This study showed that adjuvant chemotherapy for stages II-IV may be effective. However, the patients in stages II-IV who received no chemotherapy were those with poor general health or early post-operative recurrence, and the efficacy of chemotherapy for uterine sarcoma needs to be further investigated.

Regarding tumor size, which was the focus of this study, the FIGO 2008 classification uses 5 cm as the cutoff tumor size for stage I subclassification. However, Tse et al. [14] indicated that only 10-23% of tumors are ≤5 cm, with a median size of 7-9 cm, suggesting the need for size stratification for tumors >5 cm. Furthermore, Seagle et al. [15] reported a median tumor size of 9.0 cm in the National Cancer Database, and Takehara et al. [16] reported a median tumor size of 12 cm in Japan. The median tumor size was 15 cm in this study, which was much larger than 5 cm in those reports.

Several papers on tumor diameter related to sizes other than the cutoff value of 5 cm have also reported prognostic differences according to tumor size in uterine sarcoma. Several cutoff values have been established, and many studies have adopted 10 cm as the cutoff value rather than 5 cm [12,14,18,28-31]. In this study, the univariate analyses showed that OS was shorter in the group with tumor stage (III + IV) and tumor size >10 cm compared to that in the group with tumor stage (I + II) and tumor size ≤10 cm. The multivariate analysis also included age and clinical stage, with results suggesting that tumor size (≤10 cm and >10 cm) may be an important item for prognosis. In the stage I group, there were no deaths among patients with tumors ≤10 cm in size, and their prognosis was better than that for patients with tumors >10 cm. In stages II-IV, the mean OS tended to be longer for those with a tumor size ≤10 cm, although the difference was not significant. There were three patients in the stages II-IV group with tumor size ≤10 cm, including two patients with stage IV, with no deaths (OS: 11 months and 114 months, respectively). This suggests that patients with a tumor size >10 cm may possibly have a high risk of death, regardless of stage. In addition, a similar study was performed using a cutoff value of 5 cm instead of 10 cm, although the data is not shown. In that analysis, tumor size (≤5 cm vs. >5 cm) showed that OS differed significantly in the univariate analysis but not in the multivariate analysis. As indicated previously, cases of less than 5 cm are rare, and there were only three cases in this study. These results suggest that using a cutoff value of 10 cm rather than 5 cm, which has been used in the stage I subclassification, is more clinically relevant.

This study did not examine the pathological grade of mitotic index and nuclear atypia, which have been reported as prognostic factors [12,14]. Larger tumor size may potentially be influenced by higher malignant pathological, such as mitotic index and nuclear atypia, but there have been no previous reports of these associations, and future studies are warranted. While these factors are undoubtedly important, their pathology may be difficult to assess. In contrast, the tumor size examined in this study is a useful prognostic factor in clinical practice because it can be evaluated by imaging before surgery and its risk can be easily estimated. Dermawan et al. [31] reported a genomic risk stratification model for uterine leiomyosarcoma. They report that the presence of two TP53 mutations, ATRX mutation, and chr20q amplification are poor prognostic factors. They report in this literature that tumor size is an important prognostic factor that can classify risk as well as genetic mutation, using 10 cm as the cutoff value for tumor size. Thus, it is possible that the accumulation of genetic mutations may increase tumor size, leading to a more aggressive disease behavior and resistance to therapy. However, further research is needed in this regard.

This study was limited by its retrospective nature and small sample size; therefore, there may have been selection and information biases. However, this study was conducted at a single institution, making it easy to extract accurate information from the medical records and minimize the variation among the cases regarding treatment choice based on the clinical stage and other factors. In addition, the univariate and multivariate analyses identified tumor size as a factor that affected OS, which seemed to be important for uterine sarcoma risk assessment.

## Conclusions

In conclusion, this study suggests that tumor size (≤10 cm, >10 cm) may possibly have a prognostic impact on uterine sarcoma. The 2008 FIGO classification considers tumor size only in stage I; however, it was suggested that a more accurate prognosis could be estimated by considering it in stages II-IV as well. This study was limited by its retrospective nature and small sample size; therefore, the results of this study need to be further validated through future multicenter studies with larger sample sizes.
